# Bacteria commonly associated with central nervous system catheter infections elicit distinct CSF proteome signatures

**DOI:** 10.3389/fneur.2023.1102356

**Published:** 2023-02-14

**Authors:** Matthew Beaver, Dragana Noe, Ishwor Thapa, Hesham Ali, Jessica Snowden, Tammy Kielian, Gwenn L. Skar

**Affiliations:** ^1^Department of Pediatrics, University of Nebraska Medical Center, Omaha, NE, United States; ^2^Mass Spectrometry and Proteomics Core Facility, University of Nebraska Medical Center, Omaha, NE, United States; ^3^College of Information Science and Technology, University of Nebraska Omaha, Omaha, NE, United States; ^4^Departments of Pediatrics and Biostatistics, University of Arkansas for Medical Sciences, Little Rock, AR, United States; ^5^Department of Pathology and Microbiology, University of Nebraska Medical Center, Omaha, NE, United States

**Keywords:** shunt infection, central nervous system infection, biomarker, cerebrospinal fluid, proteome, *S. epidermidis*, *C. acnes*, *P. aeruginosa*

## Abstract

**Background:**

Cerebrospinal fluid (CSF) shunt infection is a common and devastating complication of the treatment of hydrocephalus. Timely and accurate diagnosis is essential as these infections can lead to long-term neurologic consequences including seizures, decreased intelligence quotient (IQ) and impaired school performance in children. Currently the diagnosis of shunt infection relies on bacterial culture; however, culture is not always accurate since these infections are frequently caused by bacteria capable of forming biofilms, such as *Staphylococcus epidermidis, Cutibacterium acnes*, and *Pseudomonas aeruginosa* resulting in few planktonic bacteria detectable in the CSF. Therefore, there is a critical need to identify a new rapid, and accurate method for diagnosis of CSF shunt infection with broad bacterial species coverage to improve the long-term outcomes of children suffering from these infections.

**Methods:**

To investigate potential biomarkers that would discriminate *S. epidermidis, C. acnes* and *P. aeruginosa* central nervous system (CNS) catheter infection we leveraged our previously published rat model of CNS catheter infection to perform serial CSF sampling to characterize the CSF proteome during these infections compared to sterile catheter placement.

**Results:**

*P. aeruginosa* infection demonstrated a far greater number of differentially expressed proteins when compared to *S. epidermidis* and *C. acnes* infection and sterile catheters, and these changes persisted throughout the 56-day time course. *S. epidermidis* demonstrated an intermediate number of differentially expressed proteins, primarily at early time points that dissipated over the course of infection. *C. acnes* induced the least amount of change in the CSF proteome when compared to the other pathogens.

**Conclusions:**

Despite the differences in the CSF proteome with each organism compared to sterile injury, several proteins were common across all bacterial species, especially at day 5 post-infection, which are candidate diagnostic biomarkers.

## Introduction

Placement of a cerebrospinal fluid (CSF) shunt is the most common pediatric neurosurgical procedure performed in the United States and is the mainstay for hydrocephalus treatment (1, 2). However, ~40,000 individuals have a CSF shunt infection annually leading to significant neurologic morbidity such as decreased intelligence quotient (IQ), school performance, seizures, and occasionally death, particularly in the pediatric population (2–10). Additionally, these infections are treatment intensive, requiring multiple surgeries to remove and replace the shunt along with long courses of parenteral antibiotics (11). Currently the diagnosis of CSF shunt infection is dependent on microbial culture of CSF from a patient with a suspected infection (12). Unfortunately, bacterial culture is not always reliable, and children are often pre-treated with antibiotics or infected with fastidious slow growing organisms like *Cutibacterium acnes (C. acnes)* or with biofilm-forming organisms like *Staphylococcus epidermidis (S. epidermidis)* or *Pseudomonas aeruginosa (P. aeruginosa)* with few metabolically active planktonic bacteria in the CSF to grow on culture (13).

When a culture is negative, diagnosis of CSF shunt infection relies on CSF indices, which are non-specific and can be difficult to interpret in the setting of recent shunt placement due to the inflammation associated with the surgery (14–17). Currently there is no rapid and accurate method of diagnosing shunt infection; therefore, there is a critical need to identify a new rapid, and accurate method for diagnosis of CSF shunt infection with broad bacterial species coverage to improve the long-term outcomes of children suffering from these infections.

Identifying biomarkers in the CSF of patients with shunt infections would significantly advance the current diagnostic paradigm and provide potential insights into the mechanisms underlying the neurologic morbidity of these infections. Our laboratory leverages its novel rat model of CNS catheter infection to interrogate CSF changes in a controlled fashion unavailable in clinical trials. Initial mass spectrometry studies in our model of *S. epidermidis* and separately of *C. acnes* infection have identified several potential biomarker candidates that discriminate between infected and sterile post-operative CSF (18, 19). However, further analysis is needed to elucidate universal biomarkers that will detect the full spectrum of bacterial pathogens responsible for shunt infection, including gram-positive, gram-negative, and anaerobic bacteria (13). Gram-negative organisms, which are the third most common cause of shunt infections, demonstrate distinct differences in inflammatory marker levels detectable in the CSF of patients with shunt infections (13, 20, 21). This is likely due to differences in the repertoire of pathogen-associated molecular patterns in the bacterial membrane, which are recognized by distinct Toll-like receptors. Nevertheless, the existence of a core CSF proteome across diverse bacterial species remains feasible given the many similar characteristics shared across pathogens. Identifying a shared CSF biomarker(s) across bacterial species would significantly advance our ability to diagnose these devastating infections. To our knowledge, there has not been a study that investigates CSF biomarkers for shunt infection across multiple bacterial species simultaneously or in a longitudinal fashion in a controlled animal model. The objective of this study was to identify both shared and distinct compounds in the CSF proteome during *S. epidermidis, C. acnes*, and *P. aeruginosa* shunt infection over time that could differentiate infection from sterile post-operative changes.

## Materials and methods

### Animals

Experiments were performed using equal numbers of 8-week-old male and female Lewis rats (Charles River Laboratories, Wilmington, MA). The Institutional Animal Care and Use Committee at the University of Nebraska Medical Center approved the protocol for animal use (protocol 16-091-09-FC) and is compliant with National Institutes of Health guidelines for the use of rodents. Animals were housed 3 to 4 per cage with species appropriate enrichment in a 12-h light-dark cycle. Food and water were provided *ad libitum*. Upon shipment, animals had a 3-day acclimation period prior to performance of any procedures. Group sizes were 5–9, which prior our studies have demonstrated is sufficient (18, 19).

### Bacterial strains and *in vitro* propagation

*S. epidermidis* 1,457 along with clinical strains of *C. acnes* and *P. aeruginosa* from the University of Nebraska Medical Center clinical microbiology laboratory were kindly provided by Dr. Paul Fey. These isolates have not been laboratory adapted or modified. A single colony of *P. aeruginosa* was inoculated into 3 ml of LB broth (Fischer Scientific, Fair Lawn, NJ) and incubated overnight with shaking at 250 rpm to achieve log phase growth. The next day the culture was diluted 1:1000 with additional LB broth and 300 μl of diluted culture was incubated overnight with the hollow silicone catheter as described below. *S. epidermidis* and *C. acnes* were propagated as previously described (18, 19, 22).

### Catheter preparation and implantation

Hollow silicone catheters (4 mm length, 1 mm diameter; Cook Medical, Inc., Bloomington, IN) were incubated overnight with either *S*. epidermidis, *C*. acnes, or *P. aeruginosa* to ensure bacterial adherence to the catheter and prevent bacterial efflux as previously described (18, 19, 22). Briefly, rats were anesthetized with inhaled isoflurane and received a subcutaneous injection of bupivacaine (1 mg/kg) and buprenorphine (0.01 mg/ kg) to mitigate any potential pain. Each animal was then positioned in a stereotactic apparatus, and a burr hole was made in the skull using a 16-gauge needle at the following coordinates to direct catheter placement into the left lateral ventricle of the brain: anterior-posterior = 21.0 mm, medial-lateral = 2.4 mm, and dorsal-ventral = 4.0 mm prior to wound closure (23).

### CSF collection

Serial CSF sampling was performed at days 1 and 5 or 14, 28, and 56 after catheter insertion. Rats were anesthetized with an intraperitoneal injection of ketamine and xylazine (87 and 13 mg/kg, respectively) and once a surgical plane of anesthetized was achieved, the back of the neck was shaved, and the animals were placed in a stereotactic apparatus as previously described (18, 19). Once positioned, the back of the neck was swabbed with betadine and ethanol then a percutaneous cisterna magna puncture was performed using a 25-gauge winged infusion set (Terumo Corporation, Somerset, NJ) with a guard to leave 4 mm of the needle tip exposed. A total of 100 to 120 μl of CSF was withdrawn for each collection and stored at −80°C until analysis.

### Bacterial enumeration from catheters and brain parenchyma

Catheters and brain tissue were collected at days 5 and 56 post-infection, as previously described, representing early and late timepoints during the infectious course (18, 19, 22). Catheters were sonicated for 5 min in 500 μl of phosphate-buffered saline (PBS) to dislodge adherent bacteria. Surrounding brain tissue from within 2 mm of the catheter tract was homogenized in 500 μl of sterile PBS supplemented with a complete protease inhibitor cocktail tablet (Roche, Basel, Switzerland) and RNase inhibitor (Promega, Madison, WI) using a Polytron homogenizer (Brinkmann Instruments, Westbury, NY). A 100 μl aliquot of brain homogenate and supernatant from sonicated catheters were used to quantitate bacterial burden by 10-fold serial dilution on blood agar plates as previously described (18, 19).

### Label-free mass spectrometry for CSF

CSF samples were processed by electrophoretic protein separation. Gels were then stained with Coomassie brilliant blue G-250 dye (Thermo Fisher) for 2 h and destained overnight in a solution of 10% acetic acid−20% methanol. Next gel pieces were excised and then washed with HPLC water and dehydrated with 100% acetonitrile (ACN). Proteins were reduced with 2 mM Tris (2-carboxyethyl)phosphine (TCEP) in 50 mM ammonium bicarbonate (NH4HCO3 [AmBic]) for 1 h at 37°C and dehydrated with ACN. Reduced proteins were alkylated with 50 mM iodoacetamide−50 mM AmBic, for 20 min in the dark with rotation. Gel pieces were dehydrated for a second time with ACN to remove all reagents. Mass spectrometry grade trypsin (15 ng/ml; Promega) was added to the samples, which were then incubation for 30 min on ice. After the removal of excess trypsin, 25 mM AmBic was added to immerse the gel pieces and incubated overnight at 37°C. Digested peptides were extracted from the gel with 50% CAN−0.1% trifluoroacetic acid solution. Samples were dried in a SpeedVac, then dissolved in 15 μl of 0.1% formic acid (FA) and subjected to liquid chromatography-tandem mass spectrometry analysis. In-gel-digested peptide samples were analyzed using an Orbitrap Fusion Lumos coupled with the UltiMate 3000 HPLC system (Thermo Scientific). Five μl (500 ng) of each sample was loaded onto the trap column (Acclaim PepMap 100, 75 μm × 2 cm; nanoViper; Thermo Scientific) using FA (0.1%) and resolved in a rapid separation liquid chromatography (RSLC) column (Acclaim PepMap RSLC; 75 μm × 15 cm; nanoViper, Thermo Scientific). Samples were eluted using a 90-min linear gradient of ACN (4 to 45%) in 0.1% FA. Parameters for all experiments were as follows: nanospray needle voltage in positive mode, 2,000 V; column flow rate, 0.3 μl/min; loading pump flow, 4 μl/min; and inject mode, μl PickUp. Orbitrap scan mode was used for MS/MS, with a resolution of 120.000 and scan range of 375 to 1,500 m/z. Peptides were placed into dynamic exclusion for 60 s after detection one time. The detector type for MS/MS was set as to Orbitrap, with a resolution of 30,000, isolation mode quadrupole (isolation window, 0.7 Da), activation type HCD (higher-energy collisional dissociation), HCD collision energy of 40%, and first mass of 110 m/z. Stainless steel emitters were purchased from Thermo Fisher (outer diameter, 150 mm; inner diameter, 30 mm; 40-mm length; inserted in a 1/32 microsleeve for installation).

### Data analysis

For mass spectrometry data every sample group (sterile, *S. epidermidis* infected, *C. acnes* infected, and *P. aeruginosa* infected) at every timepoint post-infection (days 1, 5, 14, 28, and 56 post-infection), the normalized files from the Proteomics Discoverer were processed using R programming language. For each post-infection day, the protein expression for each accession number was compared between the infected (*S. epidermidis, C. acnes*, and *P. aeruginosa*) and sterile group. The statistical significance of the differentially expressed proteins was determined using the non-parametric Wilcoxon signed-rank test in R. To identify the differentially expressed proteins unique and common to the type of infection, Venn diagrams were created using the “VennDiagram” package in R (24). For the identification of pathways enriched in the list of differentially expressed proteins, Reactome pathway database was utilized (25). The pathway analysis was performed using “ReactomePA” enrichment package in R. The heatmaps and boxplots were created using “pheatmap” and “ggplot2” packages in R (26, 27).

## Results

### CNS catheter infection kinetics

**Figure 1:**
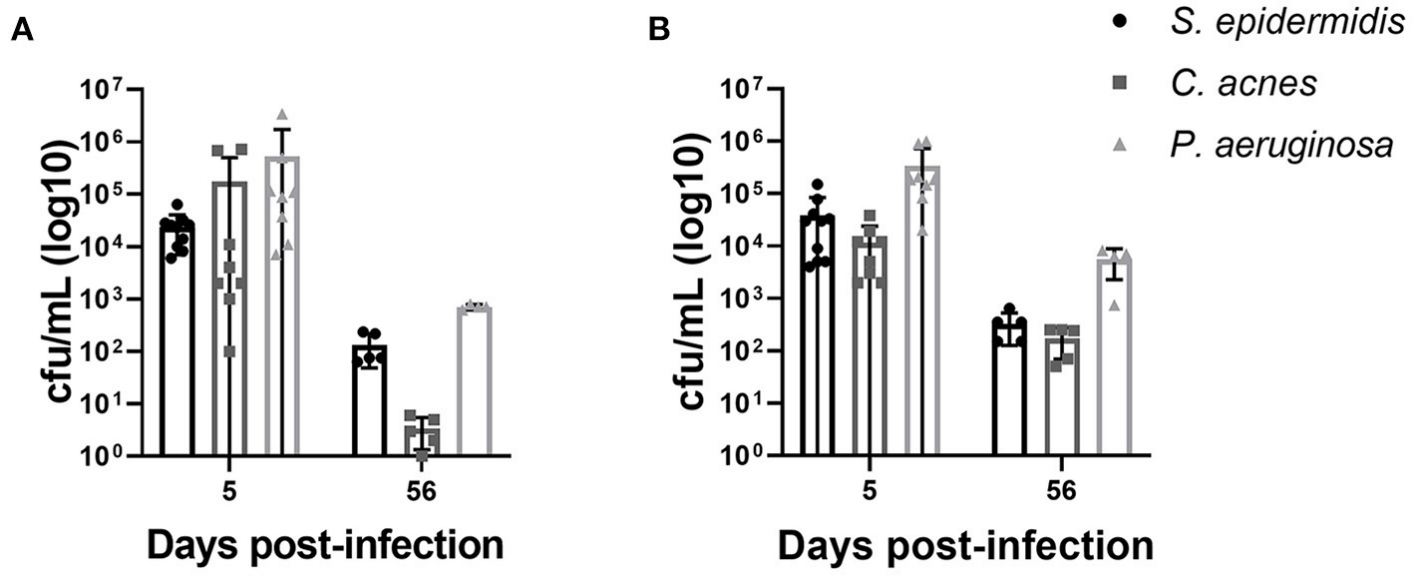
Bacterial burdens from animals infected with *S. epidermidis, C. acnes*, and *P. aeruginosa* on catheters **(A)** and from surrounding brain tissue **(B)**. Statistical analysis was not performed as bacterial burdens were quantified to demonstrate active bacterial colonization and persistence. Sterile animals had no bacterial growth in either compartment at all time points and are not shown.

We were able to reliably establish *S. epidermidis, C. acnes* and *P. aeruginosa* CNS catheter infection that recapitulates the clinical scenario of catheter associated infection with parenchymal spread and CSF changes. Using our previously published methods we were able to create a model of *P. aeruginosa* infection, which is previously undescribed in the literature to our knowledge, for comparison with *C. acnes* and *S. epidermidis* infection (18, 19). Bacterial titers from the implanted catheter and the surrounding brain tissue were recoverable at both the early and late time points in all bacterial strains demonstrating active bacterial colonization and persistence (Figure 1). Animals receiving sterile catheters did not have evidence of bacterial growth from either brain tissue or implanted catheters (data not shown).

### CSF proteome changes are influenced by bacterial strain and time post-infection

**Figure 2:**
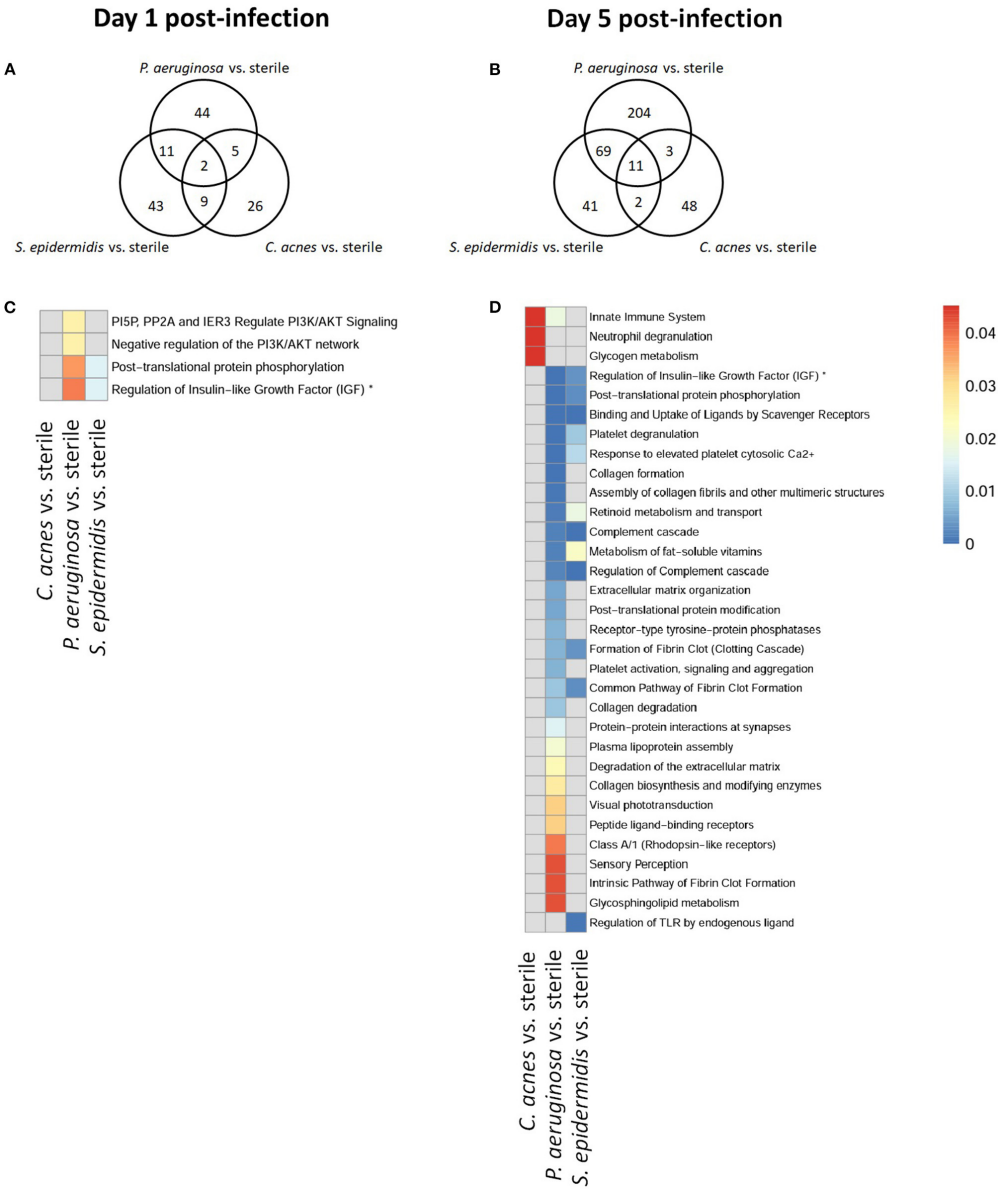
Quantity of differentially expressed proteins in *P. aeruginosa, C. acnes*, and *S. epidermidis* infection and overlap between infection types at early time points, day 1 **(A)** and 5 **(B)** post-infection as well as functional enrichment at day 1 **(C)** and 5 **(D)** post-infection. *Represents abbreviation of regulation of Insulin-like Growth Factor (IGF) transport and uptake by Insulin-like Growth Factor Binding Proteins (IGFBPS).

**Figure 3:**
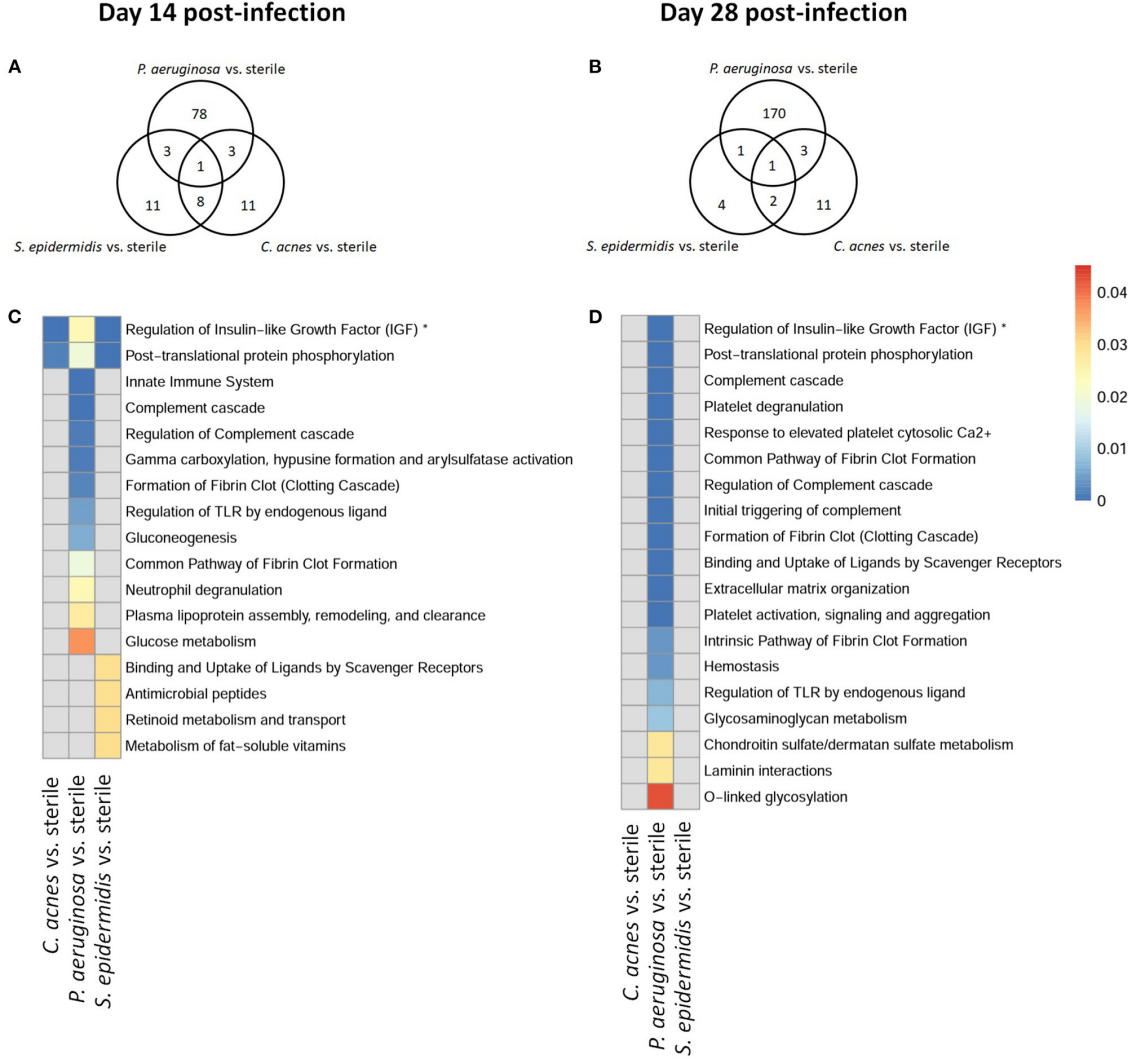
Quantity of differentially expressed proteins in *P. aeruginosa, C. acnes*, and *S. epidermidis* infection and overlap between infection types at intermediate time points, day 14 **(A)** and 28 **(B)** post-infection as well as functional enrichment at day 14 **(C)** and 28 **(D)** post-infection. *Represents abbreviation of regulation of Insulin-like Growth Factor (IGF) transport and uptake by Insulin-like Growth Factor Binding Proteins (IGFBPS).

**Figure 4:**
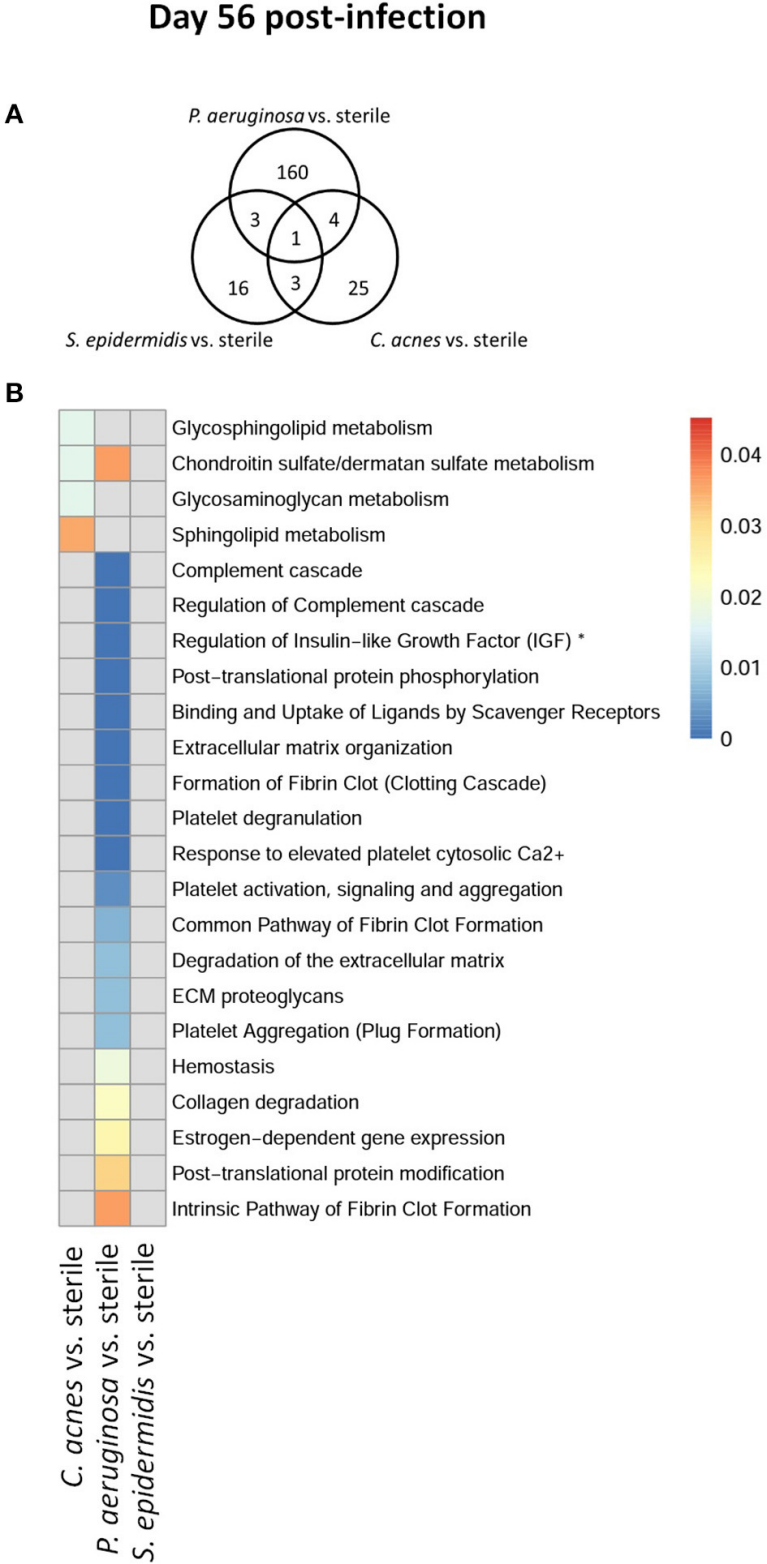
Quantity of differentially expressed proteins in *P. aeruginosa, C. acnes*, and *S. epidermidis* infection and overlap between infection types at the late time point, day 56 post-infection **(A)** as well as functional enrichment **(B)**. *Represents abbreviation of regulation of Insulin-like Growth Factor (IGF) transport and uptake by Insulin-like Growth Factor Binding Proteins (IGFBPS).

To explore whether *S. epidermidis, C. acnes*, and *P. aeruginosa*, the most prevalent bacterial pathogens associated with CSF shunt infection, each elicit a unique proteome footprint and whether this differs from post-surgical injury, serial CSF collection was performed followed by LC/MS-MS analysis of CSF. *P. aeruginosa* infection induced the greatest change in the CSF proteome, both in number of differentially expressed proteins compared to sterile catheter placement but also in terms of the length of time these changes persisted. At day 1 post-infection *P. aeruginosa* infected animals had 44 unique differentially expressed proteins compared to sterile catheters, which peaked at day 5 post-infection with 204 unique proteins (Figure 2). The number of significantly expressed proteins remained elevated compared to sterile injury at day 14 (78 proteins), day 28 (170 proteins; Figure 3) and day 56 post-infection (160 proteins; Figure 4).

Compared to *P. aeruginosa, S. epidermidis* and *C. acnes* induced significantly fewer changes in the CSF proteome over time. At day 1 post-infection *S. epidermidis* infected animals had 43 unique proteins that were differentially expressed when compared to sterile injury (Figure 2). This was similar at day 5 post-infection with 41 unique differentially expressed proteins (Figure 2). There is decreasing differential compound detection over time between sterile catheters and *S. epidermidis* infected catheters at days 14 and 28 (Figure 3). Interestingly, there is a trend of increased differentially expressed compounds in animals with *S. epidermidis* infection at day 56 compared to sterile (Figure 4). Similarly, animals with *C. acnes* infected catheters had 26, 48, and 11 uniquely detected compounds in CSF following *C. acnes* infected vs. sterile catheter placement that were not detected in either the *P. aeruginosa* or *S. epidermidis* groups (Figures 2, 3). *C. acnes* infected mice also had an increase in new compound detection, with 25 compounds detected at day 56 post-infection following infected vs. sterile catheter placements.

Despite the differences in the CSF proteome elicited by each of the bacterial pathogens compared to sterile injury, there were several shared proteins detected across all three pathogens, especially at early time points (Table 1). The number of common proteins between the different bacterial strains peaked at day 5 post-infection which corresponds to established infection. Eleven proteins were differentially expressed across all three bacterial species when compared to sterile at this time point (Table 1). Collectively, these findings have identified a subset of CSF proteins that are conserved across distinct bacterial pathogens and unique from the sterile injury response that may represent useful biomarkers to diagnose CSF shunt infections.

**Table 1:** Proteins that were differentially expressed in animals with *P. aeruginosa, S. epidermidis*, and *C. acnes* infection across all three species when compared to animals with sterile catheters at days 1, 5, 14, 28 and 56 post-infection.

**Day 1**	**Day 5**	**Day 14**	**Day 28**	**Day 56**
• Copine-1	• Zinc-alpha-2-glycoprotein	• Chitinase-3-like protein 1	• Nidogen-1	• Kininogen-1
• Septin-2	• Complement C4			
	• Complement component C6			
	• Monocyte differentiation antigen CD14			
	• Lymphocyte cytosolic protein 1			
	• Myeloperoxidase			
	• Glycogen phosphorylase, liver form			
	• 40S ribosomal protein S8			
	• Protein S100-A8			
	• Leukocyte elastase inhibitor A			
	• Similar to Vanin-3 (Predicted)			

### Kinetics of functional pathway enrichment

We further looked at which functional pathways the differentially expressed proteins corresponded to as this may have implications in the neurologic morbidity seen in association with these infections and additional biomarkers discovery. With the greatest number of differentially expressed proteins over time it was expected that *P. aeruginosa* infection demonstrated increased enrichment of functional pathways, especially late in infection when compared to *S. epidermidis* and *C. acnes* (Figures 2–4). Several pathways that were enriched throughout *P. aeruginosa* infection included those affecting immune function as well as what appears to be an injury response and wound healing at late time points. *S. epidermidis* had intermediate enrichment of pathways over time (Figures 2–4). *C. acnes* had the fewest significant pathway hits over the course of infection, although detection did start to increase at day 56 post-infection, consistent with the clinical phenotype of *C. acnes* CNS shunt infections presenting indolently (Figures 2–4) (28–31).

Functional enrichment pathway analysis revealed an increase in proteins from the complement cascade and complement regulation in both *P. aeruginosa* and *S. epidermidis* at day 5 post-infection. This confirms our previous findings with *S. epidermidis* and suggests that this may represent a conserved response to shunt infection/repair across multiple bacterial species (19). However, although complement proteins were increased in the CSF at day 14 in both *S. epidermidis* and *P. aeruginosa* infection, the kinetics of this response were distinct in that increases persisted out to day 56 in *P. aeruginosa* infected animals but not *S. epidermidis* or *C. acnes*, consistent with our prior work (19). Proteins involved in the regulation of insulin-like growth factor (IGF) transport and uptake by Insulin-like growth factor binding proteins were enriched throughout the course of infection with all three bacteria at day 14 post-infection along with proteins involved in post-translational protein phosphorylation.

## Discussion

Despite many advances in operating room protocols that have decreased shunt infection incidence, 40,000 shunt infections still occur annually. These infections cause significant short and long-term morbidity; therefore, it is essential that these infections are diagnosed and treated rapidly and accurately. In this study we have presented a model of CSF shunt infection that can directly compare the CSF proteome during infection with three of the most common causes of shunt infection to that of sterile shunt placement.

The changes in the CSF proteome during *S. epidermidis, C. acnes*, and *P. aeruginosa* seen in this study mirror the clinical scenarios seen during pediatric shunt infection (12, 21). Gram-negative infections like *P. aeruginosa* typically cause a more pronounced and early clinical presentation, where bacteria are easier to recover in culture and patients experience a severe clinical course (10, 12, 20, 32). In contrast, *S. epidermidis* typically has a milder clinical course but presents fairly early post-operatively and *C. acnes* infection is milder still and can often go undetected for months to years (12, 20, 28–31). The number and duration of proteome changes in our model correlate with clinical manifestations and laboratory findings in pediatric patients, demonstrating that this model is a powerful tool for investigating host-pathogen interactions during CSF shunt infection (12, 20, 21).

This study elucidated potential diagnostic biomarker candidates for CSF shunt infections caused by *S. epidermidis, C. acnes* or *P. aeruginosa* that commonly cause shunt infection. Complement proteins, those involved in complement regulation, and those involved in the regulation of IGF transport and uptake are possible biomarker candidates that are capable of identifying infection across multiple bacterial species. Day 5 post-infection demonstrated the highest number of biomarker candidates as there were 11 proteins that were differentially expressed across all three bacterial pathogens compared to sterile catheter placement (Table 1). As most shunt infections occur shortly after surgical procedures, this timing correlates to clinical presentation of infection, highlighting the potential of these compounds as universal biomarkers of infection. To our knowledge outside of our previous studies indicating that complement components may be useful, none of these proteins have been identified as diagnostic biomarker candidates for CSF shunt infection previously. Several of these proteins like C4, C6, monocyte differentiation antigen CD14, lymphocyte cytosolic protein, and myeloperoxidase are involved in the immune response to bacteria while others like zinc-alpha-2-glycoprotien have less clear-cut functions. However, this study highlights the potential of novel host markers of immune response for diagnosing CSF shunt infections that are not picked up on cytokine panels or that are not currently in use in routine clinical care. Additional follow up studies are needed to further test the viability of these candidates and translate our findings to clinical care.

This study demonstrated a longitudinal change in the proteome in response to all three pathogens, suggesting different phases of host-pathogen interactions. At early time points (days 1 and 5 post-infection), there were high numbers of differentially expressed proteins across all bacterial isolates. These changes are reflective of the initial host immune response and inflammatory reaction and are promising candidates for early detection of infection. Importantly, while the acute responses wane over time, there are significant changes in the CSF proteome apparent at day 56 post-infection in all pathogens, which may be reflective of the long-term neurologic morbidity that occur with these infections. The abnormalities provide valuable clues to mechanisms of long-term injury and potential pathways for mitigating the neurologic damage that occurs following shunt infections.

Upregulation of proteins in the complement cascade and regulation were observed during intermediate and late time points following *S. epidermidis* and *P. aeruginosa* infection, suggesting roles beyond pathogen control and elimination because few viable bacteria remained at these intervals. We currently have studies underway to determine the role of complement in *S. epidermidis* shunt infection. Our data also suggest that IGF and associated pathways may also participate in shunt infection as there is a significant body of data demonstrating the many functions of IGF-1 in the CNS (33–35). IGF-1 has been shown to play a role in neurogenesis, axonal development, myelination, and synapse formation and well as in neuroplasticity (33–35). Additionally, there is a growing body of literature that demonstrates the involvement of IGF-1 in neuroinflammation and its significance as a neuroprotective compound (33–36). IGF-1 promotes neuron survival as well as the repair/regenerative microglial phenotype while inhibiting the responses of astrocytes to inflammatory stimuli (36). Based on these observations, it is reasonable to infer that IGF-1 is also playing a neuroprotective and anti-inflammatory role in our model of bacterial shunt infection; however, additional studies are needed to confirm this assumption, which will be the topic of future work.

Although our model of CNS catheter infection allows us to directly compare multiple types of bacterial infection to sterile placement, it has limitations. First, the study is based on serial CSF sampling and did not allow us to examine the dynamic interplay between planktonic and biomfilm infection across the entire span of infection. We recognize that in clinical pathophysiology and in this model in particular, both planktonic and biofilm growth are present to varying degrees during the course of the infection. Therefore, these findings may be most relevant to untreated shunt infections as part of the initial diagnostic evaluation. Future study is needed to discriminate the evolution of these markers as planktonic and biofilm growth change over time. Nevertheless, these results may have correlations with human illness and can serve as the basis for future clinical studies.

With this study we have demonstrated our ability to model CSF shunt infection with multiple bacterial species that commonly cause shunt infection and are prototypical biofilm forming organisms. Not only can we establish infection, but infection appears to mimic the clinical course in pediatric patients, supporting the utility of our model for further investigations of host-pathogen interactions during shunt infection. Additionally, we demonstrated that despite the innate differences of the pathogens there are proteins that are differentially expressed across all three bacterial species when compared to sterile placement demonstrating the potential utility of CSF proteins as diagnostic biomarker candidates. These markers will be validated in future clinical studies as there are unique complexities in clinical infection including interactions between planktonic infection, biofilm, and the pediatric host over the course of infection. Through functional pathway analysis this study also revealed that the complement proteins and IGF-1 may be important in the neurologic response to shunt infection which will be followed up in dedicated studies. Understanding these differences will allow us to identify potential diagnostic biomarker candidates that are valid across multiple bacterial species and unique from the post-surgical injury response, which may provide insights into host-pathogen interactions leading to morbidity and significantly improve our ability to rapidly identify and intervene with life-altering CNS infections.

## Data availability statement

The original contributions presented in the study are publicly available. This data can be found here: ProteomeXchange Consortium, http://www.proteomexchange.org/, PXD038238.

## Ethics statement

The animal study was reviewed and approved by the Institutional Animal Care and Use Committee at the University of Nebraska Medical Center approved the protocol for animal use (protocol #16-091-09-FC) and is compliant with the National Institute of Health guidelines for the use of rodents.

## Author contributions

Experiments were designed by GS, DN, and TK. Experiments were performed by GS, MB, and DN. Data was analyzed and interpreted by GS, IT, HA, JS, and TK. Major contributors to the manuscript were GS, JS, and TK. All authors have read and approved the final manuscript.
